# ‘Blood doping’ *from Armstrong to prehabilitation:* manipulation of blood to improve performance in athletes and physiological reserve in patients

**DOI:** 10.1186/s13728-016-0046-0

**Published:** 2016-02-29

**Authors:** James O. M. Plumb, James M. Otto, Michael P. W. Grocott

**Affiliations:** Anaesthesia and Critical Care Research Unit, University Hospital Southampton NHS Foundation Trust, Southampton, UK; Integrative Physiology and Critical Illness Group, Clinical and Experimental Sciences, Faculty of Medicine, University of Southampton, University Road, Southampton, UK; Critical Care Research Area, Southampton NIHR Respiratory Biomedical Research Unit, Southampton, UK; Faculty of Medicine University of Southampton, Southampton General Hospital Mailpoint 801 South Academic Block, Tremona Road Southampton, Southampton, SO16 6YD UK; Division of Surgery and Interventional Science c/o, Institute of Sport, Exercise and Health (ISEH), 170 Tottenham Court Road, London, UK

**Keywords:** Blood doping, Blood manipulation, Anaemia, Perioperative, Surgery, Total haemoglobin mass, Autologous blood transfusion, Recombinant human erythropoietin rHuEPO, Prehabilitation, Altitude, ${\dot{\text{V}}}$O_2max_, Cycling, Hypoxia-inducible factors, Surgical outcomes

## Abstract

Haemoglobin is the blood’s oxygen carrying pigment and is encapsulated in red blood corpuscles. The concentration of haemoglobin in blood is dependent on both its total mass in the circulation (tHb-mass) and the total plasma volume in which it is suspended. Aerobic capacity is defined as the maximum amount of oxygen that can be consumed by the body per unit time and is one measure of physical fitness. Observations in athletes who have undergone blood doping or manipulation have revealed a closer relationship between physical fitness (aerobic capacity) and total haemoglobin mass (tHb-mass) than with haemoglobin concentration ([Hb]). Anaemia is defined by the World Health Organisation (WHO) as a haemoglobin concentration of <130 g/L for men and <120 g/L for women. Perioperative anaemia is a common problem and is associated with increased mortality and morbidity following surgery. Aerobic capacity is also associated with outcome following major surgery, with less fit patients having a higher incidence of mortality and morbidity after surgery. Taken together, these observations suggest that targeted preoperative elevation of tHb-mass may raise aerobic capacity both directly and indirectly (by augmenting preoperative exercise initiatives- ‘prehabilitation’) and thus improve postoperative outcome. This notion in turn raises a number of questions. Which measure ([Hb] or tHb-mass) has the most value for the description of oxygen carrying capacity? Which measure has the most utility for targeting therapies to manipulate haemoglobin levels? Do the newer agents being used for blood manipulation (to increase tHb-mass) in elite sport have utility in the clinical environment? This review explores the literature relating to blood manipulation in elite sport as well as the relationship between perioperative anaemia, physical fitness and outcome following surgery, and suggests some avenues for exploring this area further.

## Background

Haemoglobin is the blood’s oxygen carrying pigment. Erythropoietin is the hormone that stimulates human haemoglobin (and red blood corpuscle) synthesis. Its synthetic recombinant form (rHuEPO) is commonly used in clinical practice to augment haemoglobin levels, as is the use of agents that support haemoglobin synthesis (such as intravenous or oral iron, vitamin B12 or folic acid) when these are deficient. Hypoxia-inducible factor (HIF) is a transcriptional regulator that (amongst other effects) drives erythropoietin synthesis, and whereby enhances haemoglobin levels. The first recorded human blood transfusion took place in 1795 [1] and homologous blood transfusion is widely used in clinical practice for anaemic patients, including during the perioperative period. Some elite athletes have illegally tried to enhance their performance by increasing their haemoglobin levels and thereby increasing their oxygen carrying capacity via the so-called ‘blood doping’. Such activities have often taken place prior to rigorous safety trials (being properly performed for medical benefit) that are a measure of the risks that such athletes are prepared to take to achieve success [2]. For example, there is evidence that the new HIF activators are being abused within elite sport [3–6].

Aerobic capacity is defined as the maximum amount of oxygen that can be consumed by the body per unit time and is one measure of the physical fitness. ${\dot{\text{V}}}$O_2max_ is classically defined as *‘a plateau in oxygen uptake attained during maximal exercise despite further increases in exercise workload, thereby defining the limits of the cardiorespiratory system’* [7]. However, many individuals do not reach a plateau in oxygen uptake despite maximum exertion, and the term ${\dot{\text{V}}}$O_2peak_ is used instead, being the highest measured oxygen consumption during exercise, typically averaged over a 30 s period. ${\dot{\text{V}}}$O_2_ at anaerobic threshold is defined as *‘the highest sustained intensity of exercise for which the measurement of oxygen uptake can account for the entire energy requirement’.* An alternative definition is *‘the exercise intensity at which* *lactate starts to accumulate in the blood stream’* [8]. These oxygen uptake variables are in part dependent on the oxygen carrying capacity of the blood, which is in turn dependent on blood haemoglobin levels.

Anaemia is defined by the World Health Organisation (WHO) as a haemoglobin concentration of <130 g/L for men and <120 g/L for women [9]. Perioperative anaemia is common, with a quoted prevalence varying between 16 and 47 % reported in different patient cohorts (see Table 1), and is associated with increased morbidity and mortality following surgery. Using data from the European Surgical Outcomes Study (EuSOS) [10], Baron et al found that the presence of moderate anaemia was associated with a higher likelihood of in-hospital mortality than when it was absent, after adjustment for co-morbidities and the severity of the surgery [odds ratio (OR) 1.99–95 %; confidence intervals (CI) 1.67–2.37] [11]. Both ${\dot{\text{V}}}$O_2peak_ and ${\dot{\text{V}}}$O_2AT_ are positively correlated with outcome following major surgery: less physically fit patients having a higher incidence of mortality and morbidity after surgery. Much of the literature in this area is derived from studies reporting cardiopulmonary exercise testing (CPET) variables. The underlying hypothesis of these studies has been that patients with greater physiological reserve defined by CPET variables (most commonly ${\dot{\text{V}}}$O_2peak_ and ${\dot{\text{V}}}$O_2AT_) are better able to withstand the physiological challenge of surgery. Given that the oxygen uptake variables ${\dot{\text{V}}}$O_2peak_ and ${\dot{\text{V}}}$O_2AT_ are correlated with [Hb], it may be that some of the physical fitness–outcome relationship is mediated through haemoglobin related effects rather than cardiorespiratory function.

**Table 1:** Prevalence of preoperative anaemia

Study	Cohort	Study population	Prevalence of anaemia (%)
Baron—BMJ 2014 [11]	Non-cardiac, non-neurological surgery	46,539	28.7
Sagger—Anesth Analg 2013 [12]	Non-cardiac surgery	574,860	25.3
Gupta Ann Surgery 2013 [13]	Patients over 65 elective vascular surgery	31,857	47
Musallam Lancet 2011 [14]	Non-cardiac surgery	227,425	30.44
Van Straten—Circulation 2009 [15]	Cardiac surgery	10,025	16
Beattie—Anaesthesiology 2009 [16]	Non-cardiac surgery	7759	39.5
Karkouti—Circulation 2008 [17]	Cardiac surgery	3500	26
Kulier—Circulation 2007 [18]	Cardiac surgery	4804	28.1 male 35.9 female
Wu JAMA 2007 [19]	Non-cardiac surgery	310,311	42.8

Broadly based on WHO definition of anaemia, <130 g/L for men and <120 g/L for non-pregnant women

Prehabilitation is the process of enhancing functional capacity of the individual to enable him or her to withstand a subsequent stressor. This may be achieved through a single well-defined intervention (e.g. structured aerobic exercise programme) [20] or may encompass a package of smaller integrated steps leading to overall functional improvement, the so-called ‘aggregation of marginal gains’ [21–23]. Such interventions have a role in prehabilitation within clinical medicine in general, and before major surgery in particular. However, whilst the efficacy of such approaches in improving physical fitness is becoming clearer [20, 24], it is currently uncertain whether they will be effective in improving clinical outcomes in the perioperative setting. As we learn more about the relationship between physical fitness, defined by CPET-derived variables, and responses to prehabilitation in different patient groups, it may be that lessons learned from elite athletes could be applied to improving outcomes in patients around the time of surgery.

### Measuring haemoglobin concentration vs. total haemoglobin mass

Traditionally, the concentration of circulating haemoglobin [Hb] has been used as a clinical measure of the blood’s oxygen carrying capacity. However, a low [Hb] may be due to a reduced amount of haemoglobin (absolute mass of circulating haemoglobin; tHb-mass) or an increased volume of dilution (plasma volume). Thus, [Hb] may be stable and tHb-mass low in the context of acute bleeding, [Hb] normal or elevated but tHb-mass low in the context of dehydration, or [Hb] low but tHb-mass normal or high in the context of excess plasma volume (fluid). Therefore, the use of [Hb] to define blood oxygen carrying capacity may be misleading under some circumstances.

tHb-mass represents the absolute mass of circulating haemoglobin in the body, the measured [Hb] being dependent upon tHb-mass and blood volume (BV) [sum of plasma volume (PV) and total red cell volume]. The proportion of oxygen carried in solution in plasma is trivial (0.3 ml per 100 ml of plasma) under normal physiological conditions, whereas each gram of Hb binds up to 1.39 ml of oxygen. Thus, tHb-mass is the principal determinant of total blood O_2_-carrying capacity and may provide additional information regarding the clinical status of patients than that provided by [Hb] alone.

*It appears that tHb*-*mass is of greater utility in blood manipulation in elite athletes trying to improve sporting performance as it is more stable and predictable over time and also has a more direct correlation with performance. The question addressed by this review is whether tHb*-*mass, in comparison with haemoglobin concentration, is a more precise and accurate variable to guide targeting of haemoglobin manipulation, if the aim is to improve physiological reserve in patients in order to improve clinical outcomes. We also explore the techniques of blood manipulation in elite athletes and whether any of these techniques may be useful from a prehabilitation perspective within clinical medicine.*

## Haemoglobin manipulation in sport

Athletes and coaches are constantly pursuing *legal* means, such as training at altitude to augment oxygen carriage through an increase in [Hb] and thereby improving sea-level performance. However, recent revelations relating to high profile individuals within professional cycling, including Floyd Landis, Tyler Hamilton [25] and Lance Armstrong [26], have highlighted the *illegal* methods used by some athletes to improve performance, often in advance of the efforts of regulatory authorities to constrain them and of their adoption into clinical medicine [27]. It is legitimate to question whether such methods are safe (or at least fall within the broad margins of safety), if they are effective and if they could have wider applicability within clinical medicine.

Whilst a variety of agents have been used to manipulate haemoglobin levels (e.g. blood, recombinant human erythropoietin (rHuEPO), Continuous Erythropoietin Receptor Activator (CERA), hypoxia-inducible factor (HIFs) agents and possibly even ‘gene doping’ (although we do not yet have definitive evidence of this [28, 29]), the basic aim remains the same that increasing oxygen delivery (DO_2_) through elevating haemoglobin levels will augment maximum oxygen uptake (${\dot{\text{V}}}$O_2max_) and perhaps more importantly (for endurance events) increase the workload at which anaerobic threshold (AT) is reached. There is still debate around the factors that limit ${\dot{\text{V}}}$O_2max_, with candidate mechanisms including central control, cardiac limitation, mitochondrial utilisation and total oxygen delivery (the product of cardiac output and blood oxygen content). However, whilst there remains uncertainty about the dominant controlling factor, many authorities agree that in highly trained athletes, DO_2_ is a factor that contributes to ${\dot{\text{V}}}$O_2max_ limitation [30–32] and that ${\dot{\text{V}}}$O_2max_ is also, at least in part, dependent on a number of underlying genetic factors that are not amendable to modification through training [33]. Therefore, blood manipulation to augment DO_2_ has been seen as a logical, albeit illegal, approach to augmenting ${\dot{\text{V}}}$O_2max_ and thereby improving athletic performance. It is notable in this regard that tHb-mass displays a much stronger relationship with ${\dot{\text{V}}}$O_2max_ than does [Hb] [34, 35] and may therefore be a more useful marker of intervention efficacy. Here, the relationship between different physiological measures of physical fitness and performance merits consideration. Whilst the majority of sports research has focused on ${\dot{\text{V}}}$O_2peak_ or ${\dot{\text{V}}}$O_2max_ as the accepted gold standard indices of cardiorespiratory fitness, other variables may have an important role in determining performance, particularly in endurance events. As exercise increases above a threshold submaximal work rate, anaerobic respiration begins to contribute to Adenosine Triphosphate (ATP) production and this is both inefficient (relative to aerobic respiration) and unsustainable (due to progressive lactic acidosis). Therefore, when discussing performance, although a high total aerobic capacity (${\dot{\text{V}}}$O_2peak_/ ${\dot{\text{V}}}$O_2max_) is important for success in endurance sports, submaximal indices of fitness, such as the lactate or anaerobic threshold (LT/AT) and exercise efficiency/economy, may also be critical determinants of performance. For example, two athletes with the same ${\dot{\text{V}}}$O_2max_ do not necessarily perform to the same level in an endurance performance test or race: the athlete with the higher ${\dot{\text{V}}}$O_2AT_ is likely to perform better. Furthermore, the efficiency or economy with which work is done relative to energy expenditure may be important. For example, Lucia et al showed that a range of ${\dot{\text{V}}}$O_2max_ levels amongst elite cyclists could be compensated for by differences in efficiency [36]. Whilst improvements to ${\dot{\text{V}}}$O_2max_ are important, very few athletic competitions are performed at ${\dot{\text{V}}}$O_2max_ and it cannot therefore be assumed that performance will be enhanced to the same degree as ${\dot{\text{V}}}$O_2max_ increases. Intriguingly, the premise that improvement in physiological variables (i.e. aerobic capacity) enhances athletic performance (i.e. races or gold medals won) has not been well investigated. Having said that, the effects of blood manipulation on a range of physiological variables, including to ${\dot{\text{V}}}$O_2max/peak_ and ${\dot{\text{V}}}$O_2AT_, are both of relevance for athletes and may have significance in clinical contexts [37].

### What is ‘blood manipulation’/‘blood doping’?

The World Anti-Doping Agency (WADA) defines blood manipulation as the reintroduction of blood or blood products allogenic (homologous) or heterologous, the artificial enhancement of oxygen transportation or delivery and any form of intravascular manipulation of the blood or its components by physical or chemical means [3]. Blood doping is complex and rapidly evolving, as highlighted by the recent WADA amendments to the 2014 prohibited list consequent on the emergent use of Xenon and Argon as HIF activators. It was reported that Russian athletes used HIF activators at the 2014 winter Olympics in Sochi [3]. The earliest reports of ‘blood doping’ in the scientific literature date back to 1945–1947 [38, 39]. The first alleged use in elite sport was in the 1960s, when a French four times Tour de France winner (1961–1964) was named as one of the first cyclists to use the technique [40]. The first reported use in athletics comes from around the time of the 1968 Mexico City Olympic Games.

It has been said, “Increasing the oxygen transport capacity of the exercising skeletal muscles, either by means of training or doping, is the most powerful tool for improving athletic performance in aerobic sports [41]”. Below we will briefly outline some of the methods of blood manipulation used in sport.

#### Autologous blood transfusion

The link between the O_2_-carrying capacity of the blood and indices of exercise capacity such as ${\dot{\text{V}}}$O_2max_ has recently been reviewed elsewhere [34]. Haematocrit (Hct) is also known as packed cell volume (PCV) or erythrocyte volume (ECV) and is the volume percentage of red blood cells within the blood. There does not appear to be a simple linear correlation between haematocrit and increased ${\dot{\text{V}}}$O_2max_. Brun et al showed that a “low” haematocrit (Hct) (<40 %) was associated with a higher aerobic capacity [42]. However, this must be interpreted with caution, as the lowest Hct was only 36.8 % (i.e. not actually *that low*). It is probable that lower Hct levels, such as those seen in patients rather than athletes or healthy volunteers, would result in a reduced oxygen carrying capacity and therefore reduced ${\dot{\text{V}}}$O_2max_. By the 1970s, it was becoming well known that increasing tHb-mass could increase ${\dot{\text{V}}}$O_2max_. It later became clear that other factors were also important, for example, changes in diastolic function and changes in blood volume (BV) [43].

A 1982 review documented all published studies comparing exercise testing variables pre-phlebotomy, and post transfusion, at that time. It is apparent from Table 2 that a *significant* increase in [Hb] was associated with an increase in ${\dot{\text{V}}}$O_2max_. The author concluded that at least 2 units of blood were needed with frozen blood being superior to refrigerated blood [44]. Of the 14 studies in Table 2,*only 5* of them showed statistically significant improvements in [Hb] and ${\dot{\text{V}}}$O_2max_ post autologous transfusion [39, 45–48]. The results of studies failing to find such a relationship between [Hb] and exercise capacity may in part be explained by the small quantity of blood re-infused, insufficient time for the body to achieve equilibrium [Hb] after venesection, and inadequate storage of the RBCs [44].

**Table 2:** Summary of studies of blood doping and exercise

Authors	Date	Storage technique	Volume infused^a^ (ml)	Time of reinfusion post phlebotomy	Hb or Hct vs control^b^ (%)	${\dot{\text{V}}}$O_2max_ vs control (%)	End capacity^c^ vs control^b^ (%)
Pace et al	1947	Fresh^g^	2000	–	+26^d^	N.R	+34.7^d^
Gullbring et al	1960	Refridg	610	7 days	+0.7	N.R	+3
Robinson et al	1966	Refridg	1000	2 weeks	+4.8	+1.4	N.R
Ekblom et al	1972	Refridg	800	4 weeks	+2.1	+5.5^e^	+ 15.6^e^
		Refridg	1200	4 weeks	+1.3	+1.6^e^	+ 25.1^e^
Von Rost et al	1975	Refridg	900	3 weeks	+2.7	+9.0^e^	+ 37^5^
Bell et al	1976	Refridg	500	3 weeks	+1.0	+5.6^f^	+ 7.5
Ekblom et al	1976	Refridg	800	~5 weeks	+4.5^e^	+8.0^d^	N.R
Videman and RytÖmaa	1977	Refridg	4–600	2–3 weeks	+2.6	N.R	+ 3.8
Robertson et al	1978 Abst	N.R	1800	NR	N.R.	+12.8^d^	+ 15.6^d^
Williams et al	1978	Frozen	460	3 weeks	+3.3	N.R	+ 4.1
Cottrell	1979 Abst	Frozen	405	9 weeks	N.R.	~+2.0^f^	N.R
Roberston et al	1979 Abst	N.R	800	N.R.	+15.8^d^	+30.5^d^	+ 13.1^d^
Buick et al	1980	Frozen	900	7 weeks	+11^d^	+5^d^	+ 35^d^
Spriet et al	1980 Abst	Frozen	800	11 weeks	+7.9^d^	+3.9^d^	N.R
			1200	12 weeks	+10.7^d^	+6.6^d^	N.R
Williams et al	1981	Frozen	920	7 weeks	+7^d^	N.R	+2.5^d^

Reproduced with permission from Wolters Kluwer Health [44]

*NR* data not reported, *Refridg* refrigerated

^a^Whole blood or equivalent whole blood

^b^ *Control* pre-phlebotomy measurement

^c^Endurance exercise capacity, physical work capacity or performance time

^d^Statistically significant (*P* ≤ 0.05)

^e^No statistical analysis reported

^f^Predicted from submaximal exercise heart rate

^g^Fresh homologous blood; all other studies used autologous blood

In general, autologous blood transfusion seems to improve performance, but there are very few studies addressing this question directly. Improved 5-mile treadmill run times (mean improvement of 44 s) with reduced self-reported perceived exertion after autologous blood transfusion were demonstrated by Williams et al [47]. Berglund et al demonstrated a significant fall in the race times of cross-country skiers when compared to matched controls pre- and post autologous blood transfusion [49]. Brien et al took 6 well-trained runners and improved their 10 km time by an average of 1 min. Using a double-blind cross-over design, each runner received a 400 ml autologous transfusion of blood or saline repeated again 5 days apart with a 10 km race 5 days after each treatment. Five out of the 6 runners had faster race times after transfusion [50].

#### Recombinant human erythropoietin: rHuEPO

There are more data available for rHuEPO and a number of studies have shown correlation between improved performance and rHuEPO use. In 1991, Ekblom et al showed an improved ${\dot{\text{V}}}$O_2max_ post rHuEPO injection in 15 volunteers [51]. Similar results were shown by Audran et al. Table 2 from this paper shows the increase in Hct and [Hb] from day 0 to day 24 and subsequent rise in ${\dot{\text{V}}}$O_2max_ with reduction in maximum heart rate [52]. Parisotto et al attempted to develop a blood profile to detect athletes who were abusing rHuEPO and were able to demonstrate a predictable blood profile post rHuEPO usage. They measured tHb-mass (using Burge and Skinner’s method) and found a consistent increase in Hct, [Hb] and tHb-mass 3 weeks after rHuEPO administration, which persisted for 21 days. They also found a 6.3 and 6.9 % increase in ${\dot{\text{V}}}$O_2max_ compared to placebo. After a 4-week washout period, tHb-mass and ${\dot{\text{V}}}$O_2max_ had returned to baseline [53]. Birkeland et al showed in a double-blind placebo-controlled trial that injection of 5000 IU of rHuEPO thrice weekly for 4 weeks improved ${\dot{\text{V}}}$O_2max_ by 7 %. They found that Hct rose from a mean of 42.7–50.8 and peaked 1 day after rHuEPO was stopped. Haemoglobin concentration also increased in the rHuEPO group [54].

However, data supporting an improvement in performance following rHuEPO usage in athletes were still limited. Russell et al were the first to characterise the submaximal and maximal exercise adaptations to prolonged use of low dose rHuEPO. They compared 3 groups, (1) intravenous (i.v.) iron + rHuEPO, (2) oral iron + rHuEPO and (3) placebo. They performed exercise tests on a cycle ergometer at weeks 0, 4, 8 and 12. The relative increases in ${\dot{\text{V}}}$O_2max_ at weeks 4, 8 and 12 were 7.7, 9.7 and 4.5 %, respectively, for the rHuEPO + i.v. iron group; 6.0, 4.7 and 3.1 % for the oral iron + rHuEPO group; and −0.5, −0.1 and −1.0 % for the placebo group [55].

In 2007, Thomsen et al stated that “Although the positive effect of rHuEPO treatment on ${\dot{\text{V}}}$O_2max_ is clearly established, it remains unknown as to what its impact is on endurance performance”. They investigated the effect of rHuEPO on ${\dot{\text{V}}}$O_2max_ and time to exhaustion during cycle ergometry in healthy volunteers. rHuEPO significantly increased ${\dot{\text{V}}}$O_2max_ by 9.1 and 8.1 % in week 4 and 11, respectively, with no changes in the placebo group [37].

#### Emerging strategies

The range of interventions aimed at increasing tHb-mass, both in development and currently available, is large and has been extensively reviewed elsewhere [2, 4, 6, 56].

Towards the end of the 1990s, interest had grown within clinical medicine and the sporting world in using artificial oxygen carriers and perfluorocarbon emulsions [57]. However, neither has been adopted in either setting, probably due to well-recognised adverse effects and ease of detection [58].

HIF stabilisers/activators are compounds that act by mimicking hypoxia and thereby stimulating EPO synthesis via the HIF pathway. When the partial pressure of oxygen is low, HIF1α undergoes a stabilisation process, which leads to gene transcription, including that of the erythropoietin gene [59, 60]. HIF stabilisers/activators are oral compounds, which are potentially advantageous as they are simply administered and are less immunogenic than erythrocyte-stimulating agents such as rHuEPO. The number of agents available is beyond the scope of this review and has recently been summarised elsewhere [2]. There is already clear evidence of their abuse within elite sport [3–5, 56]. Cobalt is one such agent, which has adverse effects including heart, liver, kidney and thyroid toxicity as well as cancer promotion [61]. There is evidence of cobalt being abused in horse racing [62]. Xenon and argon are also both HIF activators and have both been reportedly used as performance-enhancing agents in the recent years [3, 63].

Gene therapy is also theoretically possible, but some early reports highlighting significant safety concerns including life-threatening red cell aplasia and extreme erythrocytosis have probably limited its use [64]. There are also EPO-mimetic peptides such as *Peginesatide* that are not currently in production but are nevertheless candidates for abuse [2].

### Harms: what are the downsides?

Not only is blood manipulation/doping illegal, but also many of the agents used may pose health risks to the athlete. Despite this, some athletes are apparently prepared to accept such risks to increase their chances of success. As has already been noted, manipulation of haemoglobin may be associated with a variety of adverse effects including, for example, hyper-viscosity from rHuEPO and the toxic effect of cobalt. The risks associated with blood transfusion are summarised in Table 3.

**Table 3:** Risks associated with blood transfusion and manipulation

Theoretical	Demonstrated
Age of stored blood may affect its efficacy; the so-called ‘storage lesion’ [65]	Transfer of infectious diseases [40]
	Transfusion reactions/anaphylaxis
	Increase in colorectal cancer recurrence [66]
	Phlebitis [40]
	Septicaemia [40]
	Graft versus host disease (GvHD)
	Transfusion-related immunomodulation (TRIM) [67, 68]
	Hyper-viscosity PE and DVT [40]
	Air embolism [40]
	Transfusion-related acute lung injury (TRALI)
	Risk of wrong blood (storage problems) [25]
	Detraining effect [40]
	Illegal practice to blood dope [25, 26]

## Haemoglobin manipulation in the clinical setting

Blood manipulation occurs commonly in clinical practice. In the UK, approximately 8000 units of blood are transfused each day [69] including homologous transfusion and transfusion of blood salvaged during major surgery. The level of anaemia that mandates blood transfusion is not well defined in all perioperative settings [67, 70–74] and there has been a shift over the last two decades towards more conservative transfusion strategies, particularly within intensive care [75, 76]. Whilst the association of anaemia with adverse outcome is well recognised, uncertainty remains as to whether this relationship is causal and about when and how to intervene in the perioperative period. It is unclear whether anaemia per se causes increased morbidity/mortality or whether anaemia is associated with other (perhaps unidentified) pathology, which is the cause of the adverse outcomes. Whilst the study by Baron et al suggests that anaemia alone (once all co-morbidities are corrected for) is associated with an increased mortality in perioperative patients [11], residual confounding cannot be excluded due to the observational design of this study. Of note, measures to correct anaemia (including transfusion) seem ineffective at reducing the incidence of adverse outcome. This may be because anaemia is not the cause of the underlying pathology, in which case correcting anaemia would not be expected to improve outcome, or alternatively that adverse consequences of the interventions used (such as blood transfusion) outweigh the benefits of correcting anaemia. It is commonly hypothesised that much of the morbidity associated with a more liberal transfusion strategy is due to the adverse effects of homologous stored blood rather than the increased oxygen carrying capacity actually being ineffective. The on-going evolution of preparation techniques for transfused blood is likely continuing to alter the risk–benefit ratio for different transfusion strategies. Amongst the multiple reasons for potential harm from transfused blood (see Table 3), age of the blood is an area that has recently been investigated [77]; however, no significant differences were found with regard to 90-day mortality between a fresh blood group (6.1 ± 4.9 days) and when compared with standard blood (22.0 ± 8.4 days) [77]. A recent analysis of the FOCUS study comparing a liberal and restrictive strategy in hip fracture patients found no difference in 3-year mortality [70]. The balance between the theoretical benefits of augmenting DO_2_ and the harms of transfusion remains unclear: “Blood transfusion is like a marriage: It should not be entered into lightly, unadvisedly, or wantonly, or more often than is absolutely necessary” [78].

Two large observational datasets in non-cardiac surgery have shown that anaemic patients spend more time in intensive care, suffer more complications, stay in hospital longer, consume greater hospital resource and are more likely to die [11, 14]. The same pattern is also reflected in data from cardiac surgical practice [17, 79]. NHS England has issued guidance on the management of patients who present for elective surgery. The poor outcome associated with anaemia has led to the recommendation that these patients have their surgery delayed until treatment of their anaemia has occurred [80].

Whilst anaemia is clearly important, some authors have questioned its reliability as an independent marker of ill health as it may often be linked to an underlying acute or chronic disease that may yet be undiagnosed. Interestingly, Baron et al accounted for this: after adjusting for co-morbidities and severity of the surgery, patients with moderate anaemia had a higher in-hospital mortality [odds ratio 1.99 (95 % confidence interval 1.67–2.37)] [11]. Despite this, there is currently no convincing evidence that treating the absolute value of [Hb] improves outcome.

Importantly, the majority of studies in perioperative transfusion have examined the ‘very anaemic’ and attempted to move them to the ‘slightly less anaemic’. Little work has been done to manipulate ‘normal physiology’ to target supra-optimal DO_2_ values (through Hb augmentation) in this population. Manipulation of tHb-mass, in contrast to [Hb], in patients who are about to undergo a physiological challenge, may improve their resilience to such an insult. Equating ‘performance’ gains by elite athletes to the ability of patients to survive surgery involves a substantial conceptual leap, but recent preliminary work has shown that transfusion can improve exercise variables in anaemic patients [81]. It remains uncertain whether clinical outcomes will alter alongside changes in tHb-mass and whether the closer relationship between tHb-mass (compared with [Hb]) and performance in the athletic context, will be mirrored for patient outcomes in the clinical context. Indeed it may be that the metabolic cost of modern surgery has been overestimated, thus allowing for an adequate DO2 at ‘low’ O_2_ carrying states. The recent POM-O study was a randomised controlled trial of patients undergoing major elective surgery. Patients were randomised to a postoperative protocol (fluid, with and without dobutamine) targeted to achieve their individual preoperative oxygen delivery value (goal-directed therapy) or standardised care (control). Maintaining DO_2_ appeared to be the important factor regardless of whether patients were in the goal-directed therapy group (fluids and dobutamine) or the usual care group [82].

### Prehabilitation

Interest in prehabilitation has grown off the back of the success of enhanced recovery programmes (ERPs). ERPs were set up to try improving surgical outcomes by implementing care pathways. These pathways did not focus on discovering new knowledge but placed importance on integrating the best evidence into practice. It has been shown that exercise testing in patients before surgery is feasible and that physiological gains can be made in only short periods of time [12, 83, 84], although the clinical benefits remain uncertain [24]. CPET may play a role in guiding prehabilitation [85].

In a recent study, AT improved by a mean difference of 1.2 ml kg^−1^ min^−1^ in anaemic patients who had CPET pre- and post the transfusion of autologous blood [81]. The clinical effects of an increase in ${\dot{\text{V}}}$O_2_ at the AT of around 1 ml kg^−1^ min^−1^ are uncertain and this was a small single-centre study (with consequent elevated risk of bias). However, the results were consistent with a study in thalassemic patients, showing improved exercise duration and ${\dot{\text{V}}}$O_2peak_ [86]. Pilot et al showed that autologous transfusion following hip arthroplasty improved early postoperative exercise testing variables, but this effect was equivocal by day 23. However, this study was not randomised and is therefore subject to risk of both confounding and bias [87]. This area needs further research. There is an on-going study looking at intravenous iron infusion in major abdominal surgery [88]. The transfusion trigger in major elective surgery remains unknown, particularly with regard to outcomes such as ability to mobilise postoperatively [89]. Within the hip fracture population, postoperative anaemia is associated with increased length of stay, reduced ambulation and reduced functional independence [90]. However, evidence is lacking for a liberal transfusion strategy in non-cardiac surgery ([Hb] 80–100 g/L) and this is consistent with the literature in critically unwell patients with septic shock [75].

The effects of prehabilitation training on the mitochondrial architecture, redox state and muscle capillary network remain unstudied in surgical patients and the additional effect of augmenting tHb-mass is also unknown. Training effects are of course different from the effects of blood manipulation and optimising the most critical steps in mitochondrial oxygen transport by training may be superior to using rHuEPO to enhance gene expression and induce angiogenesis; however, this remains to be elucidated. Exercise modifies mitochondrial biogenesis, not only by upregulating antioxidant enzymes but also by increasing mitochondrial number, thereby allowing for a lower level of respiratory activity for the same degree of ATP generation [91, 92]. Interestingly, Jacobs et al found that improvement in exercise performance after six sessions of high intensity training (HIT) over the span of 2 weeks was primarily attributed to enhanced oxidative potential in the skeletal muscle with no measurable effect on tHb-mass [91].

It may be that for some patients DO_2_ may be critical to VO_2_ and survival, whereas in another group of patients, anaemia, fitness levels and DO_2_ are part of a broader pattern of resilience. The biological pathways whereby regular physical activity might confer resilience include: (1) serving as a buffer against stress and stress-related disorders/chronic diseases, (2) optimising neuroendocrine and physiological responses to physical and psychosocial stressors, (3) promoting an anti-inflammatory state and (4) enhancing neuroplasticity and growth factor expression [93].

Are we then aiming for the total package of broader gains from ‘fitness’ or can we just look at specific targets such as tHb-mass? Whilst the complex biological mechanisms that relate ‘fitness’ to resilience remain opaque, it could be postulated that differences or gains (from targeted therapy or intervention) seen by patients on the CPET bike are simply a result of the other unmeasured markers of physical fitness. In the 1990s–2000s, when professional cycling had a widespread doping problem, highly trained elite athletes were experiencing significant gains over and above the increased gains in fitness from simply having a greater DO_2_ (primarily from doping, giving them a far greater tHb-mass). It is likely that patients would benefit from increased fitness levels and pilot data suggest that physical training does indeed return fitness levels back to baseline after a physiological insult such as chemotherapy [20]. Whether increasing tHb-mass offers benefit to all patients, or only those in whom it is initially subnormal remains to be elucidated.

### Future directions

We know that the manipulation of tHb-mass is possible via a number of strategies. There is good evidence that CPET variables can be improved in line with gains in tHb-mass in athletes. We also know that fitness relates to outcome following surgery. What is not known is if the same relationship between physical fitness (CPET variables) and tHb-mass exists in patients who are awaiting major surgery or critically ill in hospital. The measurement of tHb-mass and correlation to CPET variables has not been studied within clinical medicine. It is also not clear which method would be best to manipulate tHb-mass in patients and what would represent the ‘optimal’ tHb-mass in each individual, balancing the risks of an increased tHb-mass with the theoretical benefits of improved oxygen delivery. We hypothesise that tHb-mass is a more accurate variable to guide and quantify potential intervention than [Hb] as it is relatively more stable [94–97] and is not affected by changes in plasma volume which may vary greatly in the perioperative period.

Informative studies might focus on measuring tHb-mass in different patient groups and quantifying the relationship with CPET variables. Initial work to establish safety margins for different levels of haemoglobin mass in the perioperative period and the safest method for achieving them would be valuable. Preliminary studies to establish the strength of relationship between tHb-mass and outcome in comparison with that of [Hb] would also be of value. It may then be justifiable to explore whether manipulation of tHb-mass, rather than [Hb], has a positive effect on surgical outcomes in adequately powered randomised controlled trials.

It is not known what an increased tHb-mass does to mitochondrial function or if the benefits of training that come from a high intensity programme, in terms of improved mitochondrial content and function [91], can be augmented by boosting tHb-mass via rHuEPO, autologous blood transfusion or iron therapy. The ergogenic effects of EPO independent of its effect on boosting tHb-mass warrant further study and it is likely that there is a complex interplay between erythropoietin concentrations, reticulocyte migration and gene expression that may affect CPET variables and possibly outcome.

## Conclusions

In performance sport, blood doping continues to be a problem. Novel agents are continually being developed and the regulatory bodies struggle to catch up with the dopers. The success of such strategies raises the question as to whether some of these approaches may have utility in clinical practice.

In particular, the closer relationship between tHb-mass (rather than [Hb]) and ${\dot{\text{V}}}$O_2max_ raises the question as to whether we should be targeting this variable with blood manipulation techniques in the clinical setting.

The recognised association between low levels of physical fitness and adverse clinical outcomes in the perioperative context offer a specific clinical setting in which it may be valuable to address these questions. Furthermore, preoperative exercise training interventions (prehabilitation) may be optimised by such an approach.

Observational studies clarifying the relationship between tHb-mass and physical fitness and clinical outcomes in patients are required before interventional studies using this variable to target blood manipulation strategies are justified. The newer agents being used for blood manipulation in elite sport may have utility in this respect in the clinical environment.

## Health warning

Blood doping/boosting or manipulation of the blood in anyway is inherently dangerous and can result in death. The authors strongly discourage anyone from undertaking any form of blood manipulation except under the close supervision of a trained medical specialist as part of a research trial or as a planned medical intervention for ill health.

The authors would also advocate that anyone manipulating their blood within the rules of the World Anti-Doping Authorities (WADA) such as by altitude training or artificial hypobaric environment usage do so under the close supervision of a medical professional experienced in the manipulation of haemoglobin. https://www.wada-ama.org/.

## Abbreviations

AT: anaerobic threshold
BV: blood volume
CERA: Continuous Erythropoietin Receptor Activator
CPET: cardiopulmonary exercise testing
EPO: erythropoietin
ECV: erythrocyte volume
[Hb]: haemoglobin concentration
Hct: haematocrit
HIFs: hypoxia-inducible factors
LT: lactate threshold
PCV: packed cell volume
RCV: red cell volume
rHuEPO: recombinant human erythropoietin
tHb-mass: total haemoglobin mass
TRIM: transfusion-related immunomodulation
${\dot{\text{V}}}$O_2max_: maximum oxygen uptake
${\dot{\text{V}}}$O_2peak_: peak oxygen uptake
WADA: World Anti-Doping Agency
